# A primer on *in vivo* cell tracking using MRI

**DOI:** 10.3389/fmed.2023.1193459

**Published:** 2023-05-31

**Authors:** Hai-Ling Margaret Cheng

**Affiliations:** ^1^Institute of Biomedical Engineering, University of Toronto, Toronto, ON, Canada; ^2^The Edward S. Rogers Sr. Department of Electrical and Computer Engineering, University of Toronto, Toronto, ON, Canada; ^3^Ted Rogers Centre for Heart Research, Translational Biology & Engineering Program, Toronto, ON, Canada

**Keywords:** cellular imaging and cell tracking, manganese, iron oxide, gadolinium, reporter gene, ferritin

## Abstract

Cell tracking by *in vivo* magnetic resonance imaging (MRI) offers a collection of multiple advantages over other imaging modalities, including high spatial resolution, unlimited depth penetration, 3D visualization, lack of ionizing radiation, and the potential for long-term cell monitoring. Three decades of innovation in both contrast agent chemistry and imaging physics have built an expansive array of probes and methods to track cells non-invasively across a diverse range of applications. In this review, we describe both established and emerging MRI cell tracking approaches and the variety of mechanisms available for contrast generation. Emphasis is given to the advantages, practical limitations, and persistent challenges of each approach, incorporating quantitative comparisons where possible. Toward the end of this review, we take a deeper dive into three key application areas – tracking cancer metastasis, immunotherapy for cancer, and stem cell regeneration – and discuss the cell tracking techniques most suitable to each.

## Introduction

*In vivo* cell tracking refers to monitoring the localization, survival, migration, and growth of cells in a living subject via a non-invasive technology such as imaging. This capability is especially valuable in applications that involve the administration of therapeutic cells – most notably, immune cell therapy for cancer patients or stem cell therapy to regenerate healthy tissue. Once cells are introduced inside the body, their fate is influenced by a myriad of factors. Massive cell death is inevitable, but the surviving population varies greatly, depending on the cell type, injection mode, and the host tissue environment into which cells are introduced (1, 2). In many instances, therapeutic cells are also expected to home to a certain location, such as immune cells to a tumor, or to distribute evenly over a larger tissue volume. In stem cell applications, cells may be expected to differentiate and proliferate to create new tissue or repair an injury. Unfortunately, the number of surviving cells, migration to the desired therapeutic target, and extent of cell proliferation have been inconsistent, dependent partly on the patient (3) and manufacturing process (4), and can only be monitored by a non-invasive cellular imaging technique. Cell-tracking is an acknowledged, critical enabler in the development and eventual clinical translation of cell therapy.

Non-invasive cell-tracking is possible using a handful of imaging modalities – magnetic resonance imaging (MRI), positron emission tomography (PET), and optical methods. Yet, none has emerged as a front-runner due to the inherent shortcomings that accompany the unique, modality-specific advantages (Table 1). For example, optical methods offer the most versatility for labeling and visualizing cells, but limited tissue penetration constrains application to superficial tissue and small organisms such as mice (5). PET tracers provide exquisite sensitivity, but cellular toxicity from radiolabeling, tracking over hours instead of days, and poor spatial resolution severely dampen its appeal in humans (6). MRI for cell tracking suffers from low sensitivity but, otherwise, overcomes the limitations described above and can visualize cells with unlimited depth penetration and high spatial resolution (7). In this review, we examine both established and emerging MRI approaches for tracking cells *in vivo*, giving particular emphasis to the hurdles and successes intrinsic to each approach when implemented in practice. Table 2 summarizes the key points of consideration for each MRI cell labeling and tracking technique discussed in this review.

**Table 1:** Non-invasive imaging modalities amenable to cell tracking *in vivo.*

Modality	Resolution	Penetration depth	2D/3D	Sensitivity threshold to contrast agent	Signal duration	Clinically deployed?
MRI	0.1–1 mm	Unlimited	3D	10^−3^–10^−5^ M	days	Yes
PET	5–10 mm	Unlimited	3D	10^−10^–10^−12^ M	minutes	Yes
CT	0.5 mm	Unlimited	3D	10^−2^ M	days	No
Ultrasound	1 mm	Cannot pass bone/air	2D	10^0^ M	days	No
Optical	2–5 mm	< 2 cm	2D	10^−9^–10^−12^ M	days	No

**Table 2:** Comparison of MRI cell labeling and tracking methods.

	Contrast “agent”	Sensitivity	Long-term tracking	Label transfer	Multiplexing
Direct labeling	SPIOs	++++	No	Yes	No
	Gd^3+^	+++	No	No	No
	Mn^2+^ / Mn^3+^	+++	No	Yes if ionic; no otherwise	No
	^19^F agents	++	No	Yes	No
	CEST	+	No	No	Yes
Indirect labeling (reporter genes)	conventional “dark” ferritin (8)	+	Yes	No	No
	bright-ferritin (9)	+++	Yes	No	No
	DMT-1 (10)	++	Yes	No	No
	LRP (11)	+	Yes	No	Yes
	aquaporin (12)	++	Yes	No	Yes

SPIO, superparamagnetic iron oxide; Gd, gadolinium; Mn: manganese; CEST, chemical exchange saturation transfer; DMT-1, divalent metal transporter-1; LRP, lysine-rich protein.

## Direct labeling of cells

**Figure 1:**
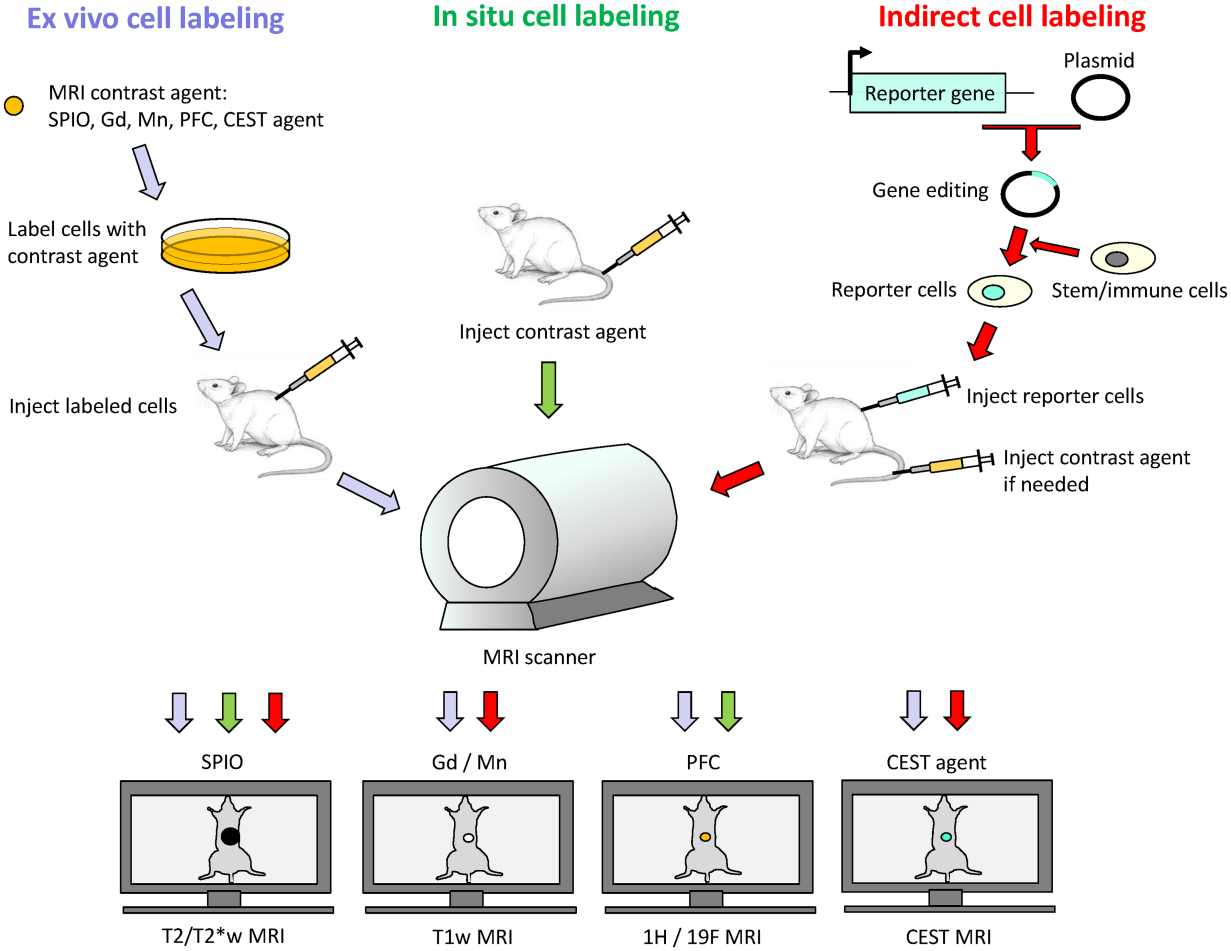
Schematic of direct and indirect labeling of cells for cell tracking on MRI. Direct labeling involves labeling cells *ex vivo* followed by cell transfer to subject, or *in situ* by delivery of contrast agent into subject for uptake by phagocytic cells. Indirect labeling involves the use of MRI reporter genes to express a desired protein in a stem cell or immunotherapy cell. These reporter cells are then transferred to the subject, and signal generation may or may not require exogenous administration of an MRI contrast agent at the time of imaging. Depending on the contrast agent used, an appropriate MRI sequence is selected: T2/T2*-weighted (T2/T2*w) MRI for iron oxide nanoparticles (SPIO), T1-weighted (T1w) MRI for gadolinium (Gd) or manganese (Mn) based contrast agents, ^1^H/^19^F MRI for perfluorocarbon (PFC) nano-emulsions, and CEST imaging for CEST-based agents.

Cell-tracking using MRI is achieved via either of two approaches: (1) *ex vivo* or *in situ* direct labeling of cells and (2) indirect labeling via MRI reporter genes (Figure 1). In this section, we review different options available for direct labeling. Indirect labeling is reviewed under the section “MRI Reporter Genes for Cell Tracking.”

### Iron oxide nanoparticles

**Figure 2:**
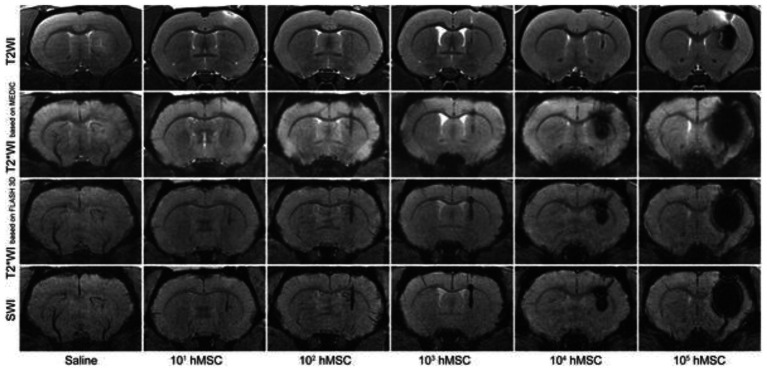
Tracking stem cells directly labeled with SPIO. Different MRI acquisitions of the rat brain injected with 20 μL saline or various quantities of SPIO-labeled human mesenchymal stem cells (hMSC) in 20 μL saline in the right striatum. All images were acquired immediately after cell transplantation. Note that T2*-weighted MRI or susceptibility weighted imaging (SWI) is needed to visualize cell numbers under 10^3^. Greater cell numbers result in severe blooming artifacts even on T2-weighted MRI. [Reproduced from *PLoS ONE*, Namestnikova D et al. “Methodological aspects of MRI of transplanted superparamagnetic iron oxide-labeled mesenchymal stem cells in live rat brain,” vol. 12, p. e0186717, 2017, under a CC BY 4.0 license http://creativecommons.org/licenses/by/4.0/].

, Iron oxide crystals coated in carbohydrates such as dextran are extremely effective agents for direct labeling of cells. The most effective particles are small ones with core diameters in the 50–180 nm range, also known as superparamagnetic iron oxide nanoparticles (SPIOs). In fact, the first cell tracking on MRI was demonstrated using SPIOs three decades ago in the early 1990s (13, 14). In these studies, blood cells were labeled by co-incubating cells with SPIO in culture media [alternatives include transfection agents or electroporation (15)]. Labeled blood cells were then readily identified by virtue of a negative contrast induced by a very strong local disturbance in the local magnetic field. Because this field disturbance extends over a volume an order of magnitude larger than that occupied by SPIOs, the area of dark contrast seen on MRI grossly overestimates that truly occupied by cells. This so-called “blooming artifact” is a double-edged sword, however: high detection sensitivity for relatively low cell numbers [10^3^ cells (16)] is achievable and desirable, but obliteration of signal in surrounding tissue precludes precise validation of cell targeting, especially in small and narrow structures of the brain, spinal cord, and heart. Nonetheless, owing to high detection sensitivity and ease of labeling, a plethora of SPIO cell-tracking applications ensued in the years and decades following, resulting in SPIO-direct labeling as the most adopted approach of all available MRI cell tracking methods. Applications spanning the tracking of immune cells (17), cancers cells (18), neural stem cells (19), cardiac stem cells (20), and smooth muscle cells (21), to name a few, abound, even today. Figure 2 illustrates tracking mesenchymal stem cells in the rat brain.

In addition to *ex vivo* cell labeling, *in situ* cell labeling is also possible with the use of ultrasmall SPIOs (USPIOs) with diameters <50 nm [which, incidentally, are not as efficacious as SPIOs for direct labeling (22)]. An intravenous injection of the iron oxide is administered to the subject, and phagocytic cells (e.g., macrophages and monocytes) will preferentially take up the iron oxide particles. In this way, inflammation foci rich with infiltrating phagocytes can be identified. Stroke, myocardial inflammation, and atherosclerosis are just a handful of many inflammatory conditions where *in situ* cell labeling with iron oxides have shown value (23–25). However, because of its non-specificity amongst different phagocytes and limited utility beyond phagocytic cells, *in situ* cell labeling remains a niche approach that confers value strictly in MRI of inflammation.

### Gadolinium-based agents

**Figure 3:**
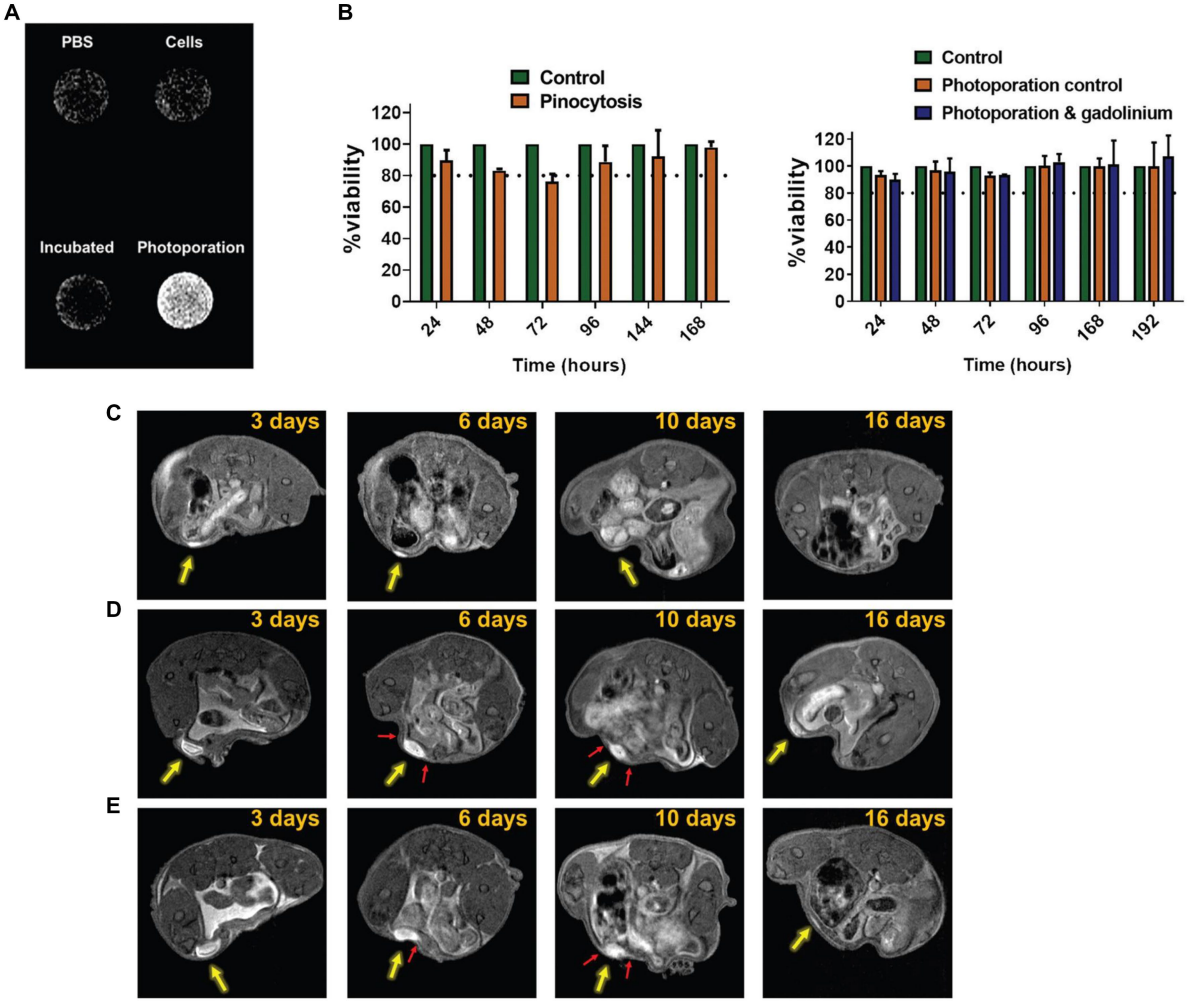
Tracking cancer cells directly labeled with Gd^3+^. Cytosolic delivery of gadobutrol via photoporation improves uptake in SK-OV-3 IP1 cells. **(A)** *In vitro* T1-weighted image of PBS, untreated cells, cells pinocytically labeled with gadobutrol, and cells labeled with gadobutrol via photoporation. **(B)** Viability assay showed no effect from photoporation or gadobutrol over 192 h. **(C–E)** *In vivo* T1-weighted cell tracking of SK-OV-3 IP1 cells labeled with gadobutrol by photoporation and injected subperitoneally (yellow arrow) for (c) 5 × 10^5^, **(D)** 4 × 10^6^, and **(E)** 8 × 10^6^ cells. Red arrows highlight the occurrence of protrusion-like structures which potentially point to the migration of the SK-OV-3 IP1 cells. [Reprinted from *Biomater Sci*, Harizaj A et al. “Cytosolic delivery of gadolinium via photoporation enables improved *in vivo* magnetic resonance imaging of cancer cells,” vol. 9, p. 4005–4,018, 2021, with permission from the Royal Society of Chemistry].

Paramagnetic gadolinium metal ions (Gd^3+^) have also been investigated for labeling and tracking cells on MRI. Methods for labeling are analogous to those for SPIOs: co-incubation, electroporation, and transfection. Unlike SPIOs, Gd-labeled cells emit a positive, or bright, contrast due to enhanced longitudinal relaxation of water. The extent of contrast enhancement is restricted to the volume occupied by labeled cells; there is no “blooming artifact” as is seen with SPIOs. Therefore, positive contrast greatly improves the precision of cell targeting and eliminates any possibility of signal obliteration in surrounding critical tissue structures. However, detection sensitivity is also lower compared to negative contrast methods, with some studies reporting 10^4^ cells (26) at the minimum detection threshold – at least 10 times higher than for SPIOs. While cell tracking with Gd-labeling has been reported for many cell types – stem cells (27), endothelial and muscle cells (21, 28), neural progenitors (29), and cancer cells (30) (Figure 3) – it is a much less common technique compared to SPIOs simply due to its lower sensitivity of detection. Large macromolecular Gd-based agents with higher relaxation efficiencies have been proposed for improving sensitivity; examples include gadolinium rhodamine dextran (31), gadolinium oxide nanoparticles (32), and gadofullerenes (33). Yet, despite these advances, Gd-based cell labeling remains relatively scant. A plausible explanation may be the cytotoxicity of Gd^3+^. As a metal foreign to the human body, Gd^3+^ has been reported to lower cell proliferation and increase reactive oxidative species (34, 35); as these are acute effects, the long-term impact on cell function remains unknown.

### Manganese-based agents

Manganese (Mn^2+^) is another paramagnetic metal ion that generates positive contrast on MRI. Many cell labeling applications involving Mn^2+^ utilize the free ionic form in MnCl_2_. Interestingly, the first application of manganese-enhanced MRI (MEMRI) was not for tracking cells but for distinguishing ischemic from healthy myocardium in dogs (36). The concept was straightforward: the divalent metal Mn^2+^ would enter viable cardiomyocytes through membrane calcium channels (37) but could not enter dead cells. Since that seminal paper, MEMRI has been used to visualize heart viability and, more commonly, neuronal connections in the brain and central nervous system (38–40).

**Figure 4:**
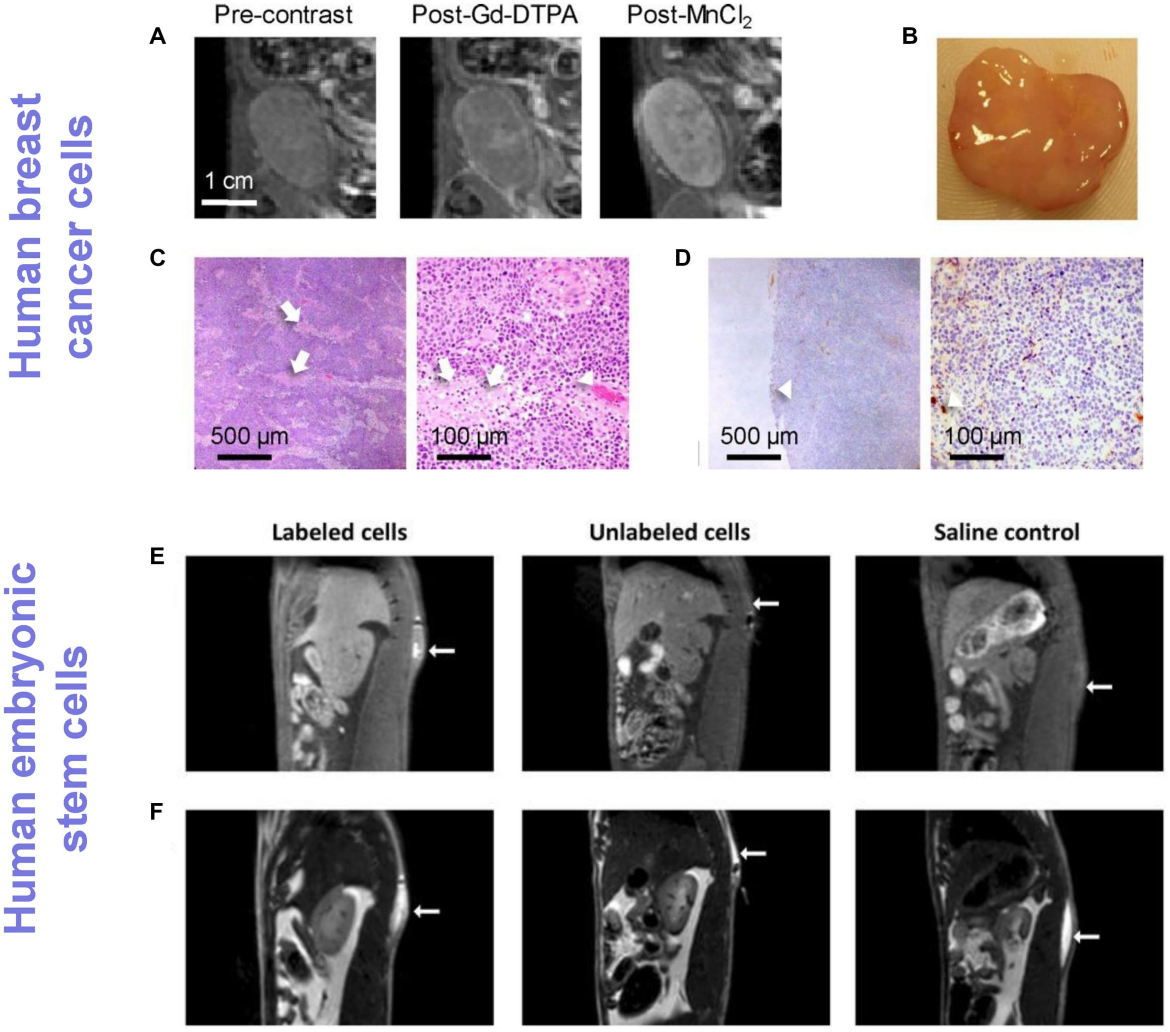
Tracking cancer cells and human stem cells directly labeled with Mn ions. **(A–D)** Human breast cancer tracking in rats and **(E,F)** human embryonic stem cell tracking in rats. **(A)** T1-weighted MRI of an orthotopic human breast ZR-75-1 cancer before and after Gd-DTPA or MnCl_2_ injection. **(B)** Gross pathology. **(C)** H&E (×4 and ×20 magnification) and **(D)** CD34 (×4 and ×20 magnification) confirmed very low vascularity (arrowheads) and patchy necrosis (arrows), which explained low vascular enhancement on Gd-DTPA but substantial cellular enhancement on MnCl_2_. [Reprinted from *J Magn Reson Imaging*, Ganesh T et al. “Manganese-enhanced MRI of minimally gadolinium-enhancing breast tumors,” vol. 41, p. 806–13, 2015, with permission from Wiley]. **(E)** T1-weighted spin-echo with fat suppression clearly shows an enhancing volume where stem cells labeled with a Mn^3+^ porphyrin were injected, whereas unlabeled cells and saline were isointense against native tissue. **(F)** T2-weighted turbo spin-echo images were acquired to localize the fluid in all injections, independent of whether or not cells were present. [Reproduced from *Sci Rep*, Venter A et al. “A manganese porphyrin-based T1 contrast agent for cellular MR imaging of human embryonic stem cells,” vol. 8, p. 12129, 2018, under a CC BY 4.0 license http://creativecommons.org/licenses/by/4.0/].

In 2006, Aoki et al. (41) reported the first cell labeling study with MnCl_2_ on human natural killer cells and cytotoxic T cells. At a labeling concentration of 0.5 mM MnCl_2_, these cells maintained their *in vitro* killing capacity and demonstrated no cytotoxicity. A few applications in direct labeling of human embryonic stem cells (42) and prostate cancer cells (43) followed in the years since. *In situ* labeling of breast cancer cells with MnCl_2_ has also been demonstrated, showing greater metal uptake in more aggressive cancer cell lines (44, 45). However, compared to labeling with SPIO and Gd^3+^ compounds, applications with MnCl_2_ are far fewer. This may be simply attributed to less fervent effort in developing Mn-containing compounds for cell labeling. Interestingly, *in situ* labeling of tumors by manganese porphyrins was discovered even earlier, in the early 1990’s. Brain tumors in animals were shown to enhance significantly against background tissue with the administration of Mn(III)TPPS (46, 47). More recently, there has been a resurgence of interest in using porphyrin as a chelator for more stable metal binding to reduce potential toxicity (48–52), as well as other formulations involving Mn^2+^ [e.g., manganese oxide nanoparticles (53, 54)]. Figure 4 illustrates the application of Mn^2+^ direct labeling of human breast cancer cells and Mn^3+^ direct labeling of human embryonic stem cells.

### Fluorine-based agents

The direct cell labeling methods described hereto all involve exploiting the inherent T2 (SPIO) or T1 (Gd^3+^ and Mn^2+^/Mn^3+^) contrast specific to the contrast agent. Unfortunately, everything in the body has a characteristic T2 and T1 contrast, which can and does appear isointense to labeled cells. This ambiguity as to the source of contrast is especially complex with T2-weighted cell tracking with SPIOs, because many endogenous sources of dark contrast exist (e.g., microbleed and hemorrhage, iron accumulation, air-tissue interface, air pockets) that are indistinguishable from labeled cells. In contrast, endogenous sources of bright T1 signal are far fewer (except for fat, which can be attenuated with fat suppression techniques).

**Figure 5:**
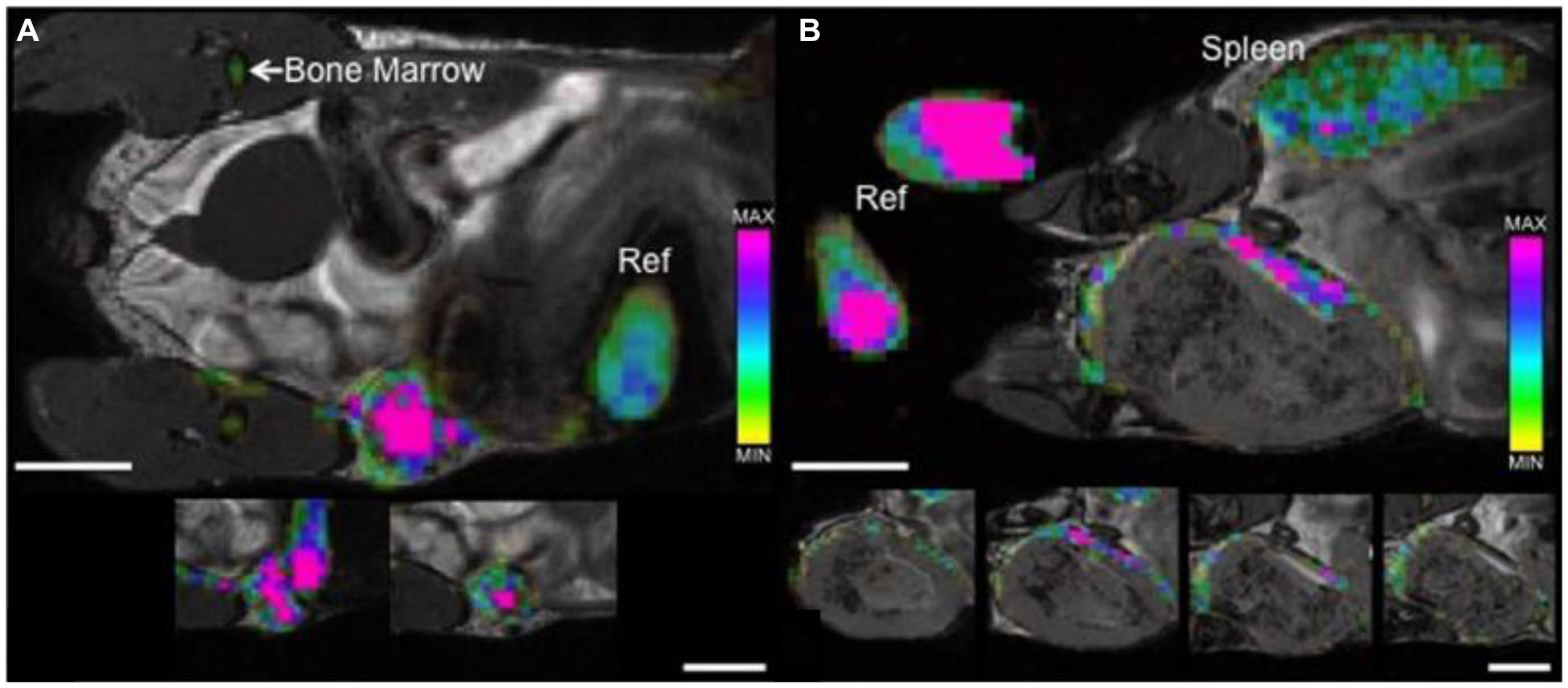
Tracking tumor-associated macrophages by direct labeling with ^19^F contrast agent. bSSFP images of mammary fat pad tumors acquired 48 h post PFC administration. **(A)** 4 days post 4 T1 cancer cell injection: ^19^F signal is detected throughout the entire tumor with a higher density visualized within the center of the tumor (pink) when compared to the periphery. **(B)** 3 weeks post 4 T1 cancer cell injection: ^19^F signal is detected, heterogeneous in density, only along the periphery of the tumor. Cropped images that are placed below each of the main images show the adjacent image slices (1 mm) containing ^19^F signal. The color bar demonstrates range of ^19^F spins, ^19^F signal is also detected in reference tubes (Ref), bone marrow and spleen. Scale bars represent 5 mm. [Reproduced from *Sci Rep*, Makela AV et al. “Quantifying tumor associated macrophages in breast cancer: a comparison of iron and fluorine-based MRI cell tracking,” vol. 7, p. 42109, 2017, under a CC BY 4.0 license http://creativecommons.org/licenses/by/4.0/].

Fluorine-based cell tracking eliminates this ambiguity entirely, as endogenous sources of mobile fluorine is well below the detection limit [< 10^−3^ μmol/g wet tissue weight (55)]. For this reason, it is preferable to metalated contrast agents for cell labeling. In 2005, Ahrens et al. (56) reported the first ^19^F-cell tracking, demonstrated for immunotherapy cells. Areas of positive contrast were readily identified as labeled cells only, and signal intensity scaled quantitatively with cell number. However, imaging with fluorine-based agents requires two separate images: ^19^F images acquired using a dedicated coil and ^1^H images acquired with a water proton coil – the former localizes cells, while the latter maps out anatomy. Because ^19^F image must be overlaid on anatomical ^1^H MRI image for cell localization, care must be taken to ensure image co-registration. This requirement for image overlay is a potential pitfall, as any mis-registration can potentially localize labeled cells in a different and wrong anatomy. Nonetheless, since the first application in 2005 ^19^F-cell tracking has been applied in monitoring inflammation (57–59), neural (60) and hematopoietic (61) stem cells, T cells in diabetes (62), and cancer cells (63). Perfluorocarbons such as perfluoropolyether and perfluoro-15-crown-5-ether are the most common ^19^F contrast agents for cell labeling (64). Figure 5 illustrates ^19^F tracking of phagocytic tumor-associated macrophages in a mouse model of murine breast cancer.

Despite the high specificity and quantitative nature of ^19^F cell tracking, sensitivity is inherently low, much lower than SPIO, Gd, or Mn-based cell tracking approaches (65). The minimum detectable cell number varies from study to study, depending on the scanner field strength, pulse sequence, and cell type. On average, the minimum detectable cell number is over 10^6^ cells per voxel (66), and this threshold barely changes even when high-SNR sequences (e.g., 3D bSSFP) are used to reduce the voxel size by 100 times, with a concomitantly longer acquisition (up to 1 h imaging) (65). From a practical standpoint, the sensitivity limit severely restricts the appeal of ^19^F cell tracking in applications where cell numbers are modest.

### CEST-based agents

**Figure 6:**
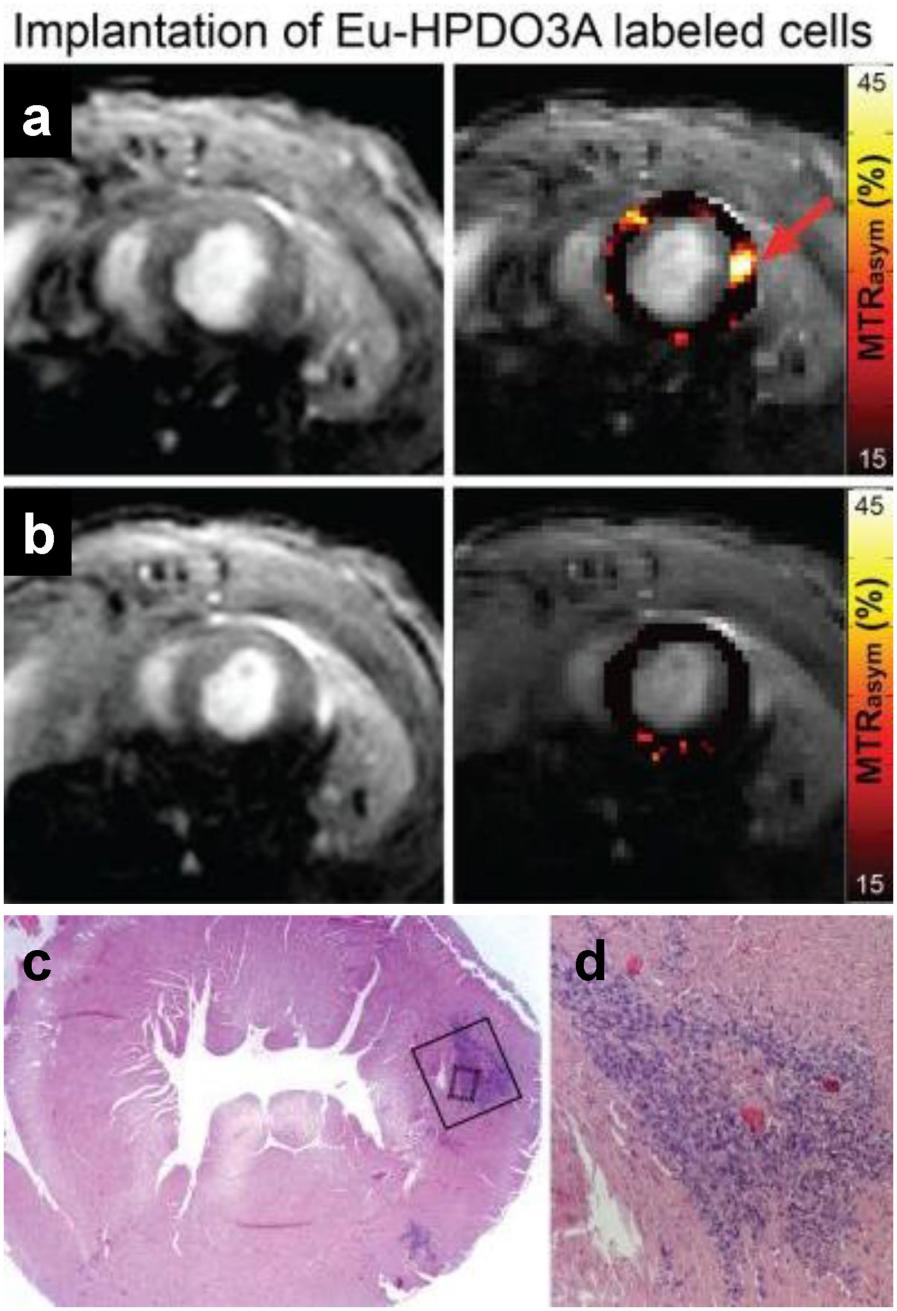
Tracking myoblasts by direct labeling with CEST contrast agent. *In vivo* CEST tracking of Eu-HPDO3A-labeled cells in the myocardium. **(A)** Location of transplanted cells (red arrow) demonstrates significantly elevated MTR_asym_ values. **(B)** Similar mapping of MTR_asym_ in an adjacent slice 2 mm toward the apex reveals absence of Eu-HPDO3A-labeled cells. All MTR_asym_ maps are displayed using a threshold of MTR_asym_ > 15%. **(C)** Hematoxylin and eosin staining of a tissue section corresponding to **(A)** reveals the presence of implanted C2C12 cells in the lateral wall of the left ventricle. **(D)** Higher magnification reveals a dense area of implanted cells (dark blue) between areas of preserved myocardium (pink). [Reprinted from *NMR Biomed*, Pumphrey A et al. “Advanced cardiac chemical exchange saturation transfer (cardioCEST) MRI for *in vivo* cell tracking and metabolic imaging,” vol. 29, p. 74–83, 2016, with permission from John Wiley & Sons].

Chemical exchange saturation transfer (CEST) is an advanced MRI method for detecting low concentrations of compounds. It achieves this by taking the compound of interest, saturating its ^1^H protons, allowing this saturation to be transferred from the compound to water, and repeating this exchange at least 100 times (to amplify water signal changes by 100 times). In the context of cell tracking, cells can be directly labeled with a CEST agent (most commonly a PARACEST agent), injected *in vivo*, and tracked over days. In this regard, it is very similar to the other direct cell labeling methods available for MRI. However, spatial resolution is poor, with voxels exceeding 2 mm in plane (67), and SNR is much lower than that for T1 or T2-weighted imaging (68), thus requiring long imaging times to boost signal above the noise floor. These limitations associated with low spatial resolution and SNR may explain the relatively sparse literature on CEST-based cell tracking. Yet, a few notable exceptions do exist: tracking of murine breast cancer (69) and myoblasts (67). Figure 6 illustrates myoblast tracking in mice.

### Limitations with exogenous cell labeling

Direct labeling of cells with an exogenous contrast agent is the most widely used cell labeling method. As seen above, a wide variety of T2, T1, and CEST-based contrast agents have shown utility for labeling and tracking cells *in vivo*. Despite ease of use and simplicity, direct labeling has several critical limitations. As alluded to previously, SPIO-induced dark contrast can be easily mistaken for endogenous dark-contrast sources, such as bleeds and air pockets, complicating the specificity of cell identification. A second limitation is the inability to distinguish living labeled cells from dead labeled cells or phagocytic immune cells that have taken up contrast agent released by dead cells (70). This limitation is seen with all MRI contrast agents, but it is especially problematic for larger particles to which macrophages have high affinity, such as SPIO and ^19^F-based agents. A third limitation is the challenge of quantifying absolute cell numbers in T2-and T1-based ^1^H imaging. For SPIO-labeled cells, there exists a linear relationship between iron concentration and cell number over a very narrow concentration range at low iron concentrations. T1-based agents, such as Gd^3+^ chelates and Mn^2+^ nanoparticles, provide a much larger linear range and at higher concentrations due to the absence of signal dropout. The fourth limitation is potential cellular toxicity from the contrast label, which is not only agent-specific but cell type-specific. While it is impossible to enumerate the safe dosing levels for all labeling agents across all cell types at different stages of maturity, literature has reported reduced endothelial cell proliferation at 0.1 mM Gd-oxide over 24 h (21), 50% neuronal cell death at 0.05 mM MnCl_2_ over 120 h (71), and reduced chondrocyte expression (72) and neural stem cell motility (73) at 25 ug/mL SPIO. It is also important to remember that even if toxicity is drastically attenuated via different chemical formulations, there has been no systematic study on their long-term stability and toxicity risks, which is an especially important consideration for Gd^3+^-based labels. In contrast, a lethal dose of perfluorocarbon has not been reported, with unaltered viability confirmed even at 20 mg/mL perfluorocarbon (74, 75). The fifth and perhaps most critical limitation is the lack of long-term cell tracking capability. As implanted cells proliferate and migrate *in vivo*, the total amount of contrast label on a per cell basis gets diluted over time. Kustermann demonstrated in murine embryonic stem cell that iron oxide-labeled cells underwent 4 replication cycles before signal diluted appreciably (76). This dilution phenomenon is observed with all direct cell labeling methods and, depending on the cell type, restricts *in vivo* cell tracking to days (18) or weeks (77) post-cell transplantation.

## MRI reporter genes for cell tracking

Reporter genes, utilized in MRI, PET, and optical imaging, provide an *indirect* method for labeling and tracking cells *in vivo*. The need for injecting or transplanting exogenously labeled cells is bypassed. Instead, the cell of interest is modified genetically through the insertion of a reporter gene that encodes proteins able to generate contrast on MRI. Because integration into the genome is required, the problem of signal dilution is effectively eliminated: so long as the genetically altered cells remain viable, a stable MRI contrast is retained regardless of cell division. This property of MRI reporter genes has attracted much attention over the past decade, providing an attractive solution to the decades-long challenge of achieving long-term and specific cell tracking.

The most published MRI reporter gene is ferritin for T2/T2* contrast (8), although a wide array of other MRI reporters has been studied over the years. Alternatives include the iron transporter MagA for T2/T2* contrast (78), divalent metal transporter-1 (DMT-1) for T1 contrast (10), LacZ for ^19^F imaging (79), lysine-rich protein (LRP) for CEST imaging (80), and human water channel aquaporin for diffusion-weighted imaging (12). Some of these methods require the exogenous administration of a MRI contrast agent to induce contrast generation, while others do not. In the following, the advantages and practical limitations of each MRI reporter are discussed.

### Ferritin

Ferritin is a universal iron-storage protein found in all mammalian cells, helping to regulate iron release in a controlled manner. Its application as an MRI reporter gene was first described in 2005 by the laboratory of Ahrens (8). An epithelial lung carcinoma cell line transfected with the ferritin transgene showed over 60x the background ferritin levels and exhibited over twice the T2 relaxation rate of wildtype control cells due to higher accumulation of supplemental iron. *In vivo*, transduced neurons and glial cells in the mouse brain displayed hypointensity on T2*-weighted MRI out to 39 days without the need for exogenous iron supplementation. Since this seminal study, no fewer than 70 reports have emerged utilizing ferritin for cell tracking. Example applications include: tracking stem cell delivery to the mouse heart (81), monitoring melanoma cells in lymph nodes in mice (82), and detecting neuronal differentiation in stem cells (83). Implementation of the ferritin method varies from study to study, with some opting for stable integration of the ferritin transgene (9, 84), some supplementing with exogenous iron to increase signal (9, 85), and some relying on endogenous iron store for contrast (81, 84).

**Figure 7:**
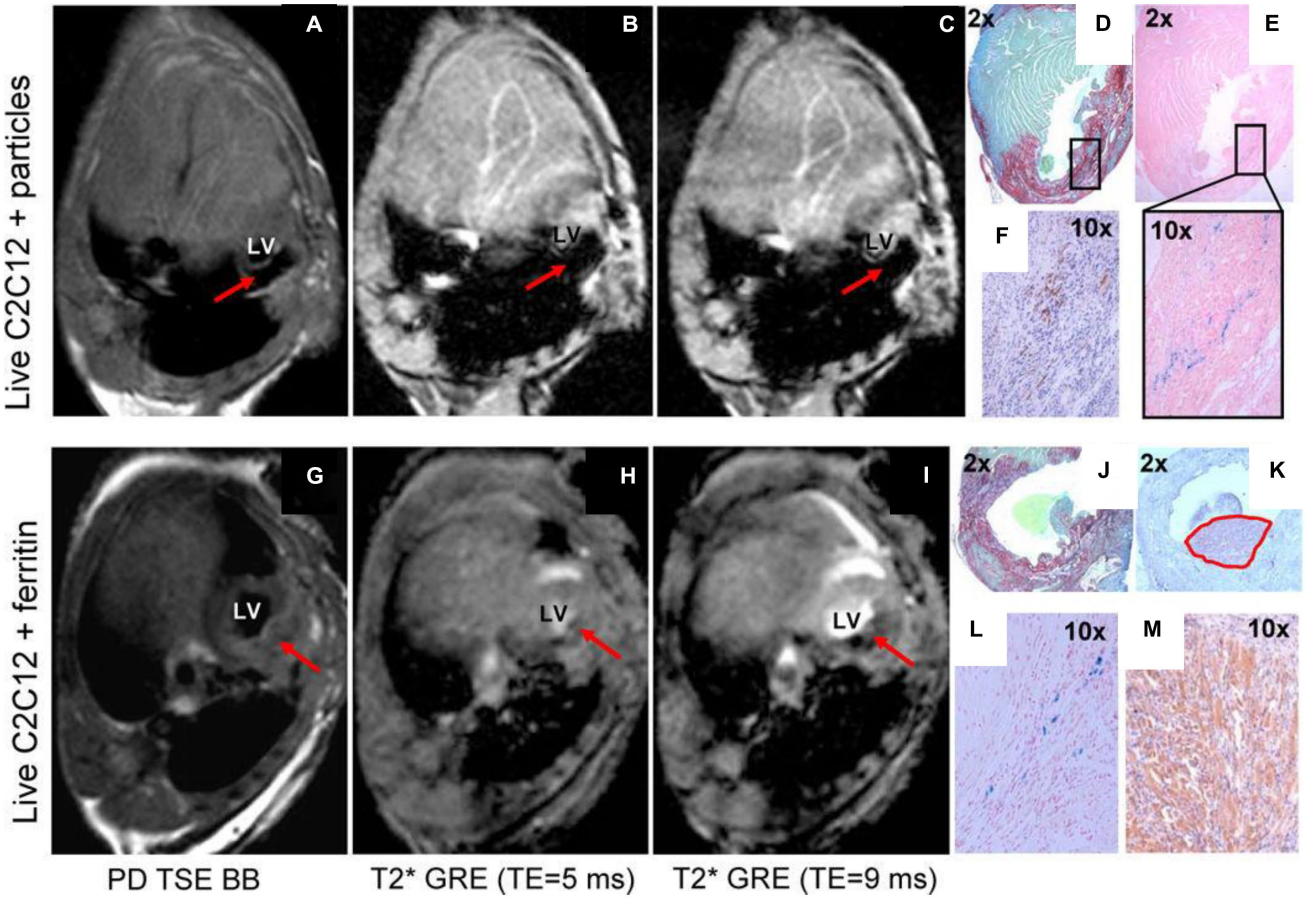
Comparison of ferritin overexpression and direct cell labeling with SPIOs. *In vivo* MRI of transplanted cells labeled by iron oxide particles **(A–F)** or transgenic C2C12 overexpressing ferritin **(G–M)**. Red arrows point to the graft area in the left ventricle of the mouse heart. **(A,G)** Proton-density weighted TSE black-blood MRI. **(B,H)** T2* GRE black-blood with TE = 5 ms. **(C,I)** T2* GRE bright-blood with TE = 9 ms. **(D,J)** Picrosirius red/fast green staining of infarct zone. (**E** with magnification, **L**) Prussian blue staining of iron accumulation. **(F,K,M)** embryonic skeletal myosin heavy chain staining to identify C2Cl2 graft. Black rectangle (in **D**) shows matching area of iron accumulation **(E)** and graft location **(F)**. Note the extensive blooming artifact in **(A–C)** compared against negligible hypointensity in **(G–I)**. [Reprinted from *J Cardiovasc Pharmacol Ther*, Naumova AV et al. “Magnetic resonance imaging tracking of graft survival in the infarcted heart: iron oxide particles versus ferritin overexpression approach,” vol. 19, p. 358–367, 2014, with permission from Sage Publications].

When ferritin overexpression is achieved via stable integration and is modest, around 2–5 times baseline, cell function and integrity is preserved, but at the cost of low sensitivity. According to some studies, the sensitivity is so low that even with exogenous iron supplementation, contrast change is negligible (9, 84). Figure 7 illustrates the much lower sensitivity of this technique compared to direct labeling with SPIOs. Furthermore, the necessity of resorting to T2*-weighted MRI to achieve sensitive cell detection is a drawback, as image distortion and low SNR are intrinsic to T2*-weighted imaging. Therefore, despite abundant evidence that ferritin overexpression is safe and non-cytotoxic, ferritin-cell tracking remains an inferior choice to direct labeling with SPIOs due to its very low sensitivity.

### Bright ferritin

**Figure 8:**
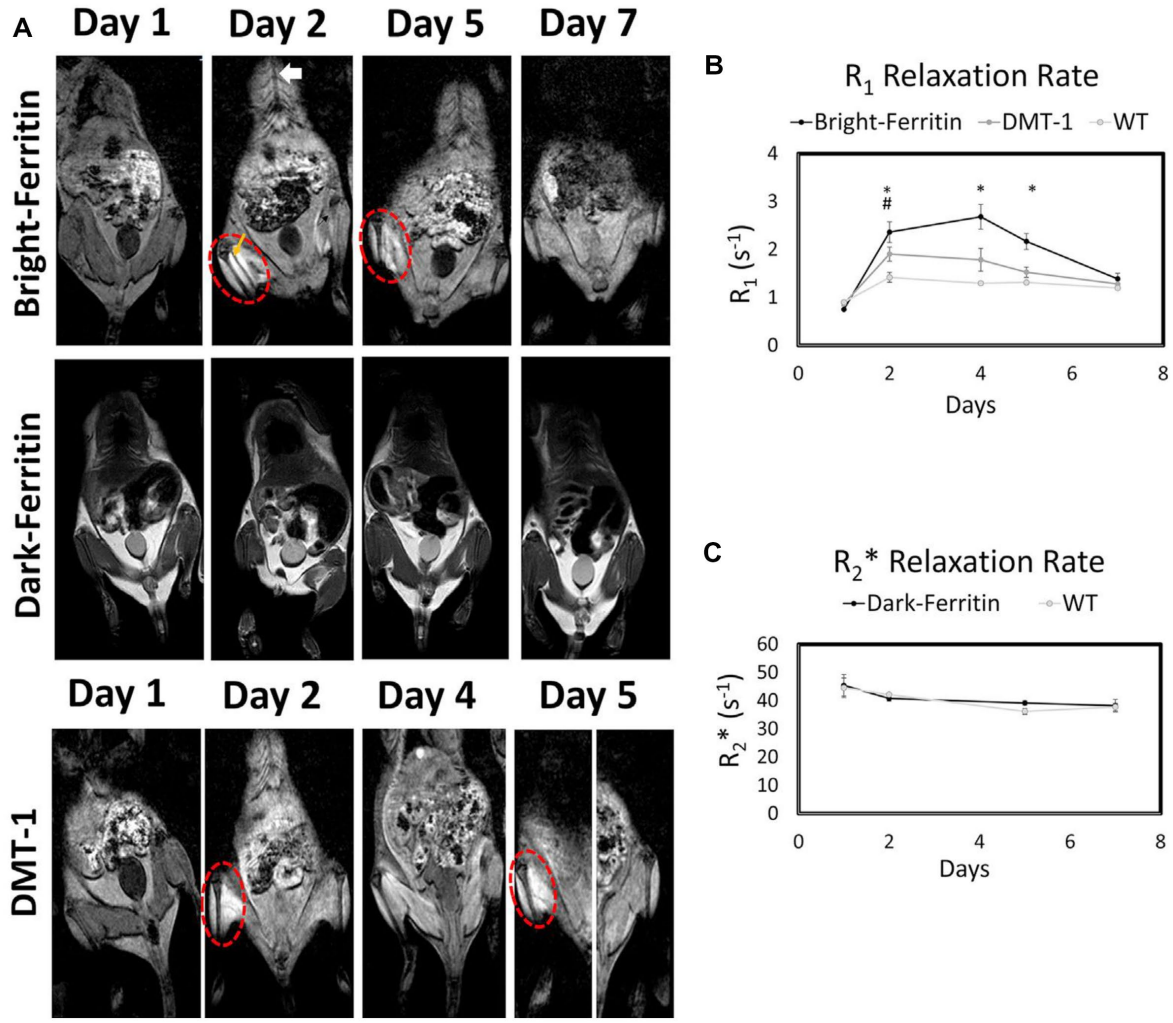
Comparison of bright-ferritin, conventional “dark” ferritin, and DMT-1 reporter genes for cell tracking. **(A)** MRI of NOD/SCID mice injected with ferritin or DMT-1 overexpressing cells in the left leg and wild-type cells in the contralateral leg (cell injection indicated by yellow arrow). MnCl_2_ supplementation (subcutaneous injection indicated by white arrow) produced large signal enhancement in the leg containing bright-ferritin (top row) and DMT-1 (bottom row) overexpressing cells. To recover signal loss in DMT-1 cells after 4 days, MnCl_2_ was re-applied to turn “on” signal. Dark-ferritin cells (middle row) showed no contrast change, both without (day 1 and 2) and with iron supplement (day 5 and 7); oral iron supplementation was given daily after day 2. Quantitative relaxometry revealed **(B)** significant changes in R1 in the bright-ferritin and DMT-1 legs relative to wild-type but **(C)** minimal difference in R2* on conventional dark-ferritin imaging. Difference in R1 between bright-ferritin and DMT-1 is significant at all times (*p < 0.05); difference in R1 between DMT-1 and wild-type is significant only at day 2 (#*p* < 0.05). Data are represented as mean ± SD. [Reprinted from *iScience*, Szulc DA et al. “Bright ferritin – a reporter gene platform for on-demand, longitudinal cell tracking on MRI,” vol. 23, p. 101350, 2020, with permission from Elsevier].

In 2020, Szulc et al. reported a highly sensitive, T1-weighted MRI approach to overcome the low sensitivity of conventional “dark” ferritin-based cell tracking (9). In that study, human embryonic kidney (HEK) cells were stably transfected with the ferritin transgene, and a modest two-fold protein overexpression was attained. No impact on cell viability, proliferation, and metabolism was observed at this low overexpression level. Both *in vitro* and *in vivo* mouse experiments confirmed that ferritin-overexpressing cells, when exposed to supplemental MnCl_2_, exhibited a bright contrast and large T1 reduction on MRI, sustained for 5 days. Chemistry analysis and microscopy revealed the formation of manganese-ferritin nanoparticles inside cells and their eventual degradation by normal cellular pathways. This study not only uncovered Mn^2+^ as an alternative metal to iron that could be sequestered in ferritin protein, but also boasted much higher sensitivity, SNR, and image quality from utilizing T1-weighted MRI. Ongoing investigation of the “bright-ferritin” platform shows promising results in the tracking of human embryonic stem cells and differentiated cardiomyocytes for cardiac therapy (86). Figure 8 compares the contrast and longevity of signal amongst three methods: bright-ferritin, conventional dark ferritin with and without iron supplementation, and DMT-1, to be discussed next.

### Divalent metal transporter-1

The divalent metal transporter-1 (DMT-1) is a plasma membrane protein that transports ferrous iron and some, not all, divalent metals ions across the plasma membrane (87). Metals that are transported by DMT-1 include manganese, cobalt, copper, and zinc; however, calcium, also a divalent metal, is not (88, 89). While most studies of DMT-1 revolve around understanding iron transport and metabolism, Bartelle et al. were the first to report its use for indirect cell tracking (10). A number of different cell lines, including HEK cells and murine glioma cells, were stably transfected to overexpress DMT-1 by up to 3-fold. This overexpression of DMT-1 was accompanied by an over 3-fold increase in T1 relaxation rate *in vitro* when 0.3 mM MnCl_2_ was supplemented in culture media for 1 h. As the authors noted, the observed change in T1 relaxivity was greater than the 1.8-fold increase in T2 relaxation rate from labeling ferritin-overexpressing HEK cells at 1 mM Fe for 24 h (90). Consistent with the higher efficiency of DMT-1 relative to ferritin coupled with iron, *in vivo* results in glioma cells implanted in mice confirmed a bright contrast was sustained relative to control cells beyond 24 h.

A handful of DMT-1 cell tracking papers have since been published after the 2013 discovery, focused on tracking experimental HEK or human stem cells *in vivo* (9, 91). Both studies demonstrated a clear demarcation of bright contrast where the cells resided. In the HEK cell tracking study in mice, it was further demonstrated that bright signal could be *recalled* on demand with a simple administration of MnCl_2_ (9). This demonstration is one of the rare literature evidence where MRI signal from remaining viable reporter cells can be recalled, as needed, in a longitudinal fashion – what is dubbed true “long-term” cell tracking. Interestingly, the authors also compared the bright contrast obtained from DMT-1 against that from bright ferritin: they discovered that the latter provided a T1 relaxivity of 17.7 mM^−1^ s^−1^, where DMT-1 provided only half the relaxivity at 7.1 mM^−1^ s^−1^.

### Metal-free reporter genes

The MRI reporter genes described thus far all involve metallic substances that are either paramagnetic or superparamagnetic. This is limiting in that only one cell type can be tracked, precluding the possibility of monitoring multiple labeled cell populations as found in fluorescent methods. In 2007, Gilad et al. described the concept of transfecting cells with a lysine-rich protein (LRP) encoding vector (11). Radiofrequency irradiation at the amide proton frequency results in exchange with water protons (i.e., CEST effect). LRP overexpression showed no toxicity on viability or metabolic rate. *In vivo* mouse MRI at 11.7 Tesla demonstrated an 8% increase in CEST signal in LRP-overexpressing xenografts, whereas control xenografts exhibited a 3% increase in CEST signal. This approach is attractive in that additional frequency-selective reporters may be designed to label different cell populations. Nonetheless, as with all CEST-based method, low SNR remains a substantial barrier to widespread adoption.

A more sensitive, metal-free alternative is changing cellular water permeability. Mukherjee et al. introduced in 2016 a class of MRI reporters based on the human water channel, aquaporin 1 (12), to increase water movement into and out of a cell. No impact on cell viability was noted in the array of cells stably transfected to overexpress aquaporin 1. Water diffusivity increased (i.e., diffusion-weighted signal decreased) across the cell membrane by at least 2-fold for all cell types tested, with no change in T1 or T2 relaxation times, which implies an orthogonal channel for MRI. However, the use of diffusion-weighted MRI also introduces confounding influences from other sources of negative diffusion-weighted contrast. A second reporter gene that also modulates transmembrane water transport is the urea transporter (UT-B). Similar to aquaporin 1, UT-B expression can be increased to effect a proportional elevation in the apparent water exchange rate (92).

### Limitations with MRI reporter genes

All MRI reporter gene cell tracking methods suffer from lower sensitivity of detection compared to direct cell labeling methods. In some instances, the contrast change may not even be detectable, as with iron-supplemented ferritin overexpressing cells. However, it is important to recognize that research in advancing MRI reporter genes has seen only a fraction of the effort applied to direct cell labeling methods. Therefore, many of the current limitations, as discussed in the following, may be surmounted in the not-too-distant future.

Low sensitivity of detection often results from inadequate *in vivo* exposure to a MR-active agent. For example, when ferritin-overexpressing cells are supplemented with iron, or when ferritin-overexpressing or DMT-1-overexpressing cells are supplemented with Mn^2+^, the *in vivo* bioavailability of either iron or Mn^2+^ is naturally much lower than it would be in an *in vitro* direct labeling setting. If one could increase the metal dosing systemically, sensitivity would automatically go up. Yet, this is an impractical solution, as achieving the required dose at the target site can easily lead to toxic overdosing in other organs. A potential solution is to administer the metal supplement locally to the site of interest, but this has limited value once cells have migrated away from the initial injection site.

Potential cytotoxicity or adverse impact on cell function is another concern with gene editing. All the MRI reporters in the literature involve changing metal homeostasis or water diffusion into and out of a cell. At low overexpression of reporter genes, most studies have confirmed that cells are unaffected. At high levels of overexpression, the sensitivity gained is offset by potential toxic effects. The balance between maintaining normal cell function and achieving higher sensitivity of detection is delicate, and it must be determined individually on different cell types and at different stages of maturity.

Finally, there is the risk of gene silencing, a rarely considered topic in MRI cell tracking. Gene silencing refers to the reduced expression of a gene. In the context of MRI reporter genes, a transgene is inserted into the genome with the hope that all the progeny can be tracked on MRI. However, that may not be the case. For instance, if one were to create a stable ferritin-overexpressing stem cell line, they may find that the ferritin transgene is silenced as the cells undergo differentiation and multiple passages. In this way, the “long-term” cell tracking capability that sets MRI reporter gene methods apart, is eventually lost. A recent report described how using CRISPR/Cas9 technology to insert their transgene into the AAVS1 safe harbor locus of human induced pluripotent stem cells did not sustain stable expression (93). In fact, gene silencing occurred during cardiomyocyte differentiation, leading to a decrease in expression from 98.9 to 1.3%. Checking for potential gene silencing is a must for any researcher working with MRI reporter genes.

## Cell tracking in the preclinical and clinical space

Cell therapy occupies a center stage in today’s innovative therapeutics and can be loosely divided into those involving stem cells (for regeneration) or therapeutic cells (for treating cancer or metabolic disorders) in patients. Preclinically, the applications are more numerous, and include the investigation of tumor development, cancer metastasis, and anti-tumor therapies. Cell tracking can help answer questions related to: Where do the cells go? How many cells survive? How long do cells survive? Do cells replicate and migrate? Does cell persistence contribute to tissue functional and/or structural recovery? As researchers, one of the most pressing questions we ask is which cell tracking technique is most suitable for the application at hand. In the following, we will explore this question for the three broad applications of cell tracking.

### Studying cancer metastasis

One of the critical gaps in cancer research is an incomplete understanding of cancer metastasis and how different cancers respond to treatment. The question as to why cancers return in some patients but not in others has led to the concept of cancer stem cells, namely, that sub-population of cells capable of self-renewal and tumorigenicity. While the cancer stem cell population has not been probed specifically in MRI studies, many papers over the past two decades have described tracking the metastasis of cancer cells in experimental animal models to better understand what organs are susceptible (94–96). However, the majority of these studies employ SPIOs, which has limited durability and suffer from signal dilution, especially in rapidly proliferating cancer cells (97). An alternative approach, which has been rarely reported, is *in situ* labeling of metastatic cancers with MnTPPS, which showed bright contrast in both the primary rat brain tumor and the solitary metastasis (98). Another viable alternative to studying cancer progression and treatment response is to utilize MRI reporter genes that can provide a much longer lasting contrast necessary for studying the migration of metastatic cancer cells. This is an unexplored area of research, and care must be exercised in selecting an MRI reporter gene that does not alter the tumorigenic properties of the specific cancer cell type. Characterization of the relevant baseline protein expression levels is also necessary in the cancer cell of interest.

### Monitoring stem cell therapy

Unlike cancer cells, stem cells cannot thrive in inhospitable tissue environments, undergoing massive cell death in the hours and days after transplantation *in vivo*. A conservative estimate amongst stem cell researchers is a minimum 90% death rate, but over 99% cell death has also been reported in 2000 (99). However, with the availability of immune-compromised animals, drugs for immunosuppression, and new cell delivery vehicles, *in vivo* cell survival can be improved by an order of magnitude (100) – even 20% cell survival has been reported in the mouse spinal cord 4 weeks after stem cell transplantation (101). Amongst the surviving cell population, cell number will gradually scale up over the course of weeks and even months, but not necessarily to an appreciable number. Furthermore, some cell types, such as cardiomyocytes, do not replicate, and other cell types, such as neural progenitors, will migrate over a larger tissue volume. These nuances complicate cell tracking on MRI, because a low cell number and cell density can be expected, possibly falling below the limit of detection.

One strategy to ensure high detection sensitivity when massive cell death occurs initially is to use a direct cell labeling method. Small molecule-based labeling is recommended for this purpose, and large molecular structures (e.g., SPIOs and ^19^F nano-emulsions) should be avoided to avert the transfer of label from dying injected cells to phagocytic cells. This strategy should, at minimum, provide a more truthful depiction of cell survival and distribution in the early days after cell transplantation. Nonetheless, with all exogenous tracers, whether they are small molecules or large nanostructures, the possibility still exists that tracer outside of the intended cell target is being detected.

In the longer term, MRI reporter gene methods are the only option for monitoring proliferation from the remaining viable cell population and tracking their migration in the body. However, given that reporter gene methods generally suffer from low sensitivity, signal may or may not return, depending on the abundance and distribution of proliferating cells. Another key consideration is the anatomy into which cells are injected. If the anatomy consists of small, narrow structures (e.g., spinal cord, thin myocardial walls, renal cortical layers), then a dark-contrast method may obliterate the signal of surrounding critical anatomy. In this case, a positive-contrast reporter gene, such as DMT-1 or bright-ferritin, is extremely valuable. On the other hand, if therapeutic cells are delivered to a more homogenous tissue where less precise targeting can be tolerated (e.g., brain, liver), dark-contrast reporter genes, such as those modulating water permeability or iron sequestration in ferritin, may be exploited.

### Tracking cancer immunotherapy

**Figure 9:**
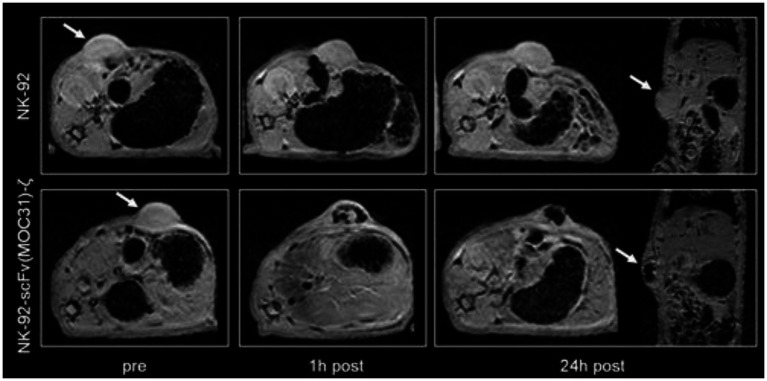
Tracking cancer T-cell therapy with SPIO direct labeling. Axial and coronal T2*-weighted gradient echo images (TR = 500 ms, TE = 5.1 ms; FA = 30°) of two representative EpCAM-positive DU145 tumors before, at 1 h and at 24 h post-injection (p.i.) of ferumoxides-labeled parental NK-92 cells (arrows, upper row) and of ferumoxides-labeled EpCAM-targeted NK-92-scFv (MOC31)-ζ cells (arrows, lower row). A marked negative tumor enhancement is noted at 1 and 24 h p.i. of the NK-92-scFv (MOC31)-ζ treated tumor. By contrast, there is no signal change at 1 or 24 h after injection of the ferumoxides-labeled parental NK-92 cells. [Reprinted from *Magn Reson Med*, Meier R et al. “Depicting adoptive immunotherapy for prostate cancer in an animal model with magnetic resonance imaging,” vol. 65, p. 756–763, 2011, with permission from Wiley].

Broadly speaking, immune cells are readily tracked *via in situ* cell labeling with SPIOs or ^19^F nano-emulsions. These labeling methods are particularly useful, because they label cells circulating *inside* the body, but the downside is lack of distinction amongst different types of immune cells. With novel cancer immunotherapy, or more specifically T-cell transfer therapy, immune cells are taken from the patient, modified in the lab to attack the cancer more effectively, and re-administered to the patient. To verify tumor homing, long-term viability, retention, expansion, and absence of off-target effects, modified T-cells must be easily differentiated from other immune cells. This requirement stipulates the replacement of non-specific *in situ* labeling with either a direct or indirect labeling method specific to T-cells. Direct labeling with SPIOs is the most straight-forward approach, but simple incubation may be ineffective, as T-cells are non-phagocytic and do not take up extracellular particles readily. For this reason, a transfection agent can be employed to increase uptake. Furthermore, as T-cells are expected to distribute sparsely after injection, a high label content must be achieved on a per cell basis. Therefore, strategies to achieve high labeling efficiency without compromising T-cell function are crucial. Early reports in this domain described SPIO-direct labeling of cytotoxic T lymphocytes in a mouse glioblastoma model (102) and adoptive cell therapy in a dog prostate cancer model (103) (Figure 9). A more ideal, albeit less sensitive, alternative is MRI reporter gene for T-cell tracking, but no literature report exists. Finally, a quantitative cell tracking method is highly desirable in this application, as optimization of T-cell therapy requires not only correct targeting but also delivering the correct number of cells. In this sense ^19^F or T1-weighted imaging methods may be more suitable than T2/T2*-based tracking methods.

## Utilizing cell tracking in different applications

No single cell tracking approach suits all applications, and despite the limitations of each method, abundant flexibility remains for selecting the most effective cell tracking method in any application. For example, applications that require only immediate confirmation of cell homing are best suited to high-sensitivity direct labeling approaches. If massive cell death is anticipated, then it is best to avoid iron oxides particles for direct labeling, as their uptake by phagocytic cells is well known. On the other hand, if the background host tissue into which cells are transplanted is fairly uniform in contrast (e.g., brain), then iron oxide labeling provides the highest sensitivity without being confused for dark-susceptibility contrast that arises in many abdominal organs. One can also envision multiplexing different approaches to reap the strengths of each approach. For example, it is conceivable to directly label cells that express a certain MR reporter gene: the direct label provides high sensitivity in the days following cell transplantation, while the reporter gene can be exploited for longer term cell tracking in the weeks and months following. Table 3 summarizes the pros and cons of some of the most effective cell tracking methods described in this primer and suggests applications to which each is amenable.

**Table 3:** Suggested applications of select MRI cell labeling and tracking methods.

Method	Pros	Cons	Suggested Applications
Exogenous labeling with SPIOs	Highest sensitivity of cell detection	Short-term labeling Signal obliteration of nearby tissue structures Label transfer to phagocytic cells Ambiguity if other dark contrast is present	Stem cell engraftment in the brain and liver Homing of immune cells to tumors
Exogenous labeling with Mn^2+^/Mn^3+^ agents	High sensitivity of cell detection High specificity of cell detection Useful in small tissue spaces	Short-term labeling	Stem cell engraftment in the brain, spinal cord, heart, lungs, liver, kidneys, and skeletal muscle Primary tumor engraftment
Exogenous labeling with CEST agents	Multiple cell types can be tracked simultaneously	Low sensitivity of detection Lower spatial resolution	Tracking engraftment of multiple cell types
Endogenous labeling with “Bright ferritin”	Longitudinal cell tracking High specificity to only viable cells	Medium sensitivity of cell detection Requires cell transfection with reporter gene	Long-term stem cell survival in the heart, kidneys, and skeletal muscle Cancer metastasis
Endogenous labeling with DMT-1	Longitudinal cell tracking High specificity to only viable cells	Medium sensitivity of cell detection Requires cell transfection with reporter gene	Long-term stem cell survival in the brain, heart, liver, and skeletal muscle

SPIO, superparamagnetic iron oxide; Mn, manganese; CEST, chemical exchange saturation transfer; DMT-1, divalent metal transporter-1.

## Conclusion

*In vivo* cell tracking is an indispensable player in the development of next-generation stem cell regeneration and immune therapy, and in our understanding of cancer metastasis and developmental biology. Over the past three decades, an impressive array of MRI cell tracking approaches has been demonstrated. Direct labeling approaches were the first to emerge and remain the most commonplace, given their ease of implementation and high sensitivity. Indirect labeling methods (via MRI reporter genes), on the other hand, hold the potential to true “long-term” tracking of only surviving cells and their progeny. However, these methods are less sensitive, and their optimization remains a work-in-progress. Regardless of the approach taken, labeled cells must not be affected adversely (i.e., morphology, function, stemness, differentiation capacity, etc. remain intact), while sensitivity is maximized as much as is reasonable. The ideal cell labeling approach is highly unique to the application and may differ not only for different cell types but also for different anatomical targets.

## Author contributions

The author confirms being the sole contributor of this work and has approved it for publication.

## Funding

This work was supported by Medicine by Design Pivotal Study Fund (grant #MbDPEFR1-2021-04); Natural Sciences and Engineering Research Council of Canada (grant #2019-06137); Canadian Institutes of Health Research (grant #PJT 175131); Canada Foundation for Innovation/Ontario Research Fund (grant #34038); New Frontiers Research Fund (grant #NFRFE-2020-00509); and a Dean’s Spark Professorship from the University of Toronto.

## Conflict of interest

The author declares that the research was conducted in the absence of any commercial or financial relationships that could be construed as a potential conflict of interest.

## Publisher’s note

All claims expressed in this article are solely those of the authors and do not necessarily represent those of their affiliated organizations, or those of the publisher, the editors and the reviewers. Any product that may be evaluated in this article, or claim that may be made by its manufacturer, is not guaranteed or endorsed by the publisher.
